# Fast-Forming Dissolvable Redox-Responsive Hydrogels: Exploiting the Orthogonality of Thiol–Maleimide and Thiol–Disulfide Exchange Chemistry

**DOI:** 10.1021/acs.biomac.2c00209

**Published:** 2022-06-13

**Authors:** Ismail Altinbasak, Salli Kocak, Rana Sanyal, Amitav Sanyal

**Affiliations:** † Department of Chemistry, Bogazici University, Bebek, Istanbul 34342, Turkey; ‡ Center for Life Sciences and Technologies, Bogazici University, Bebek, Istanbul 34342, Turkey

## Abstract

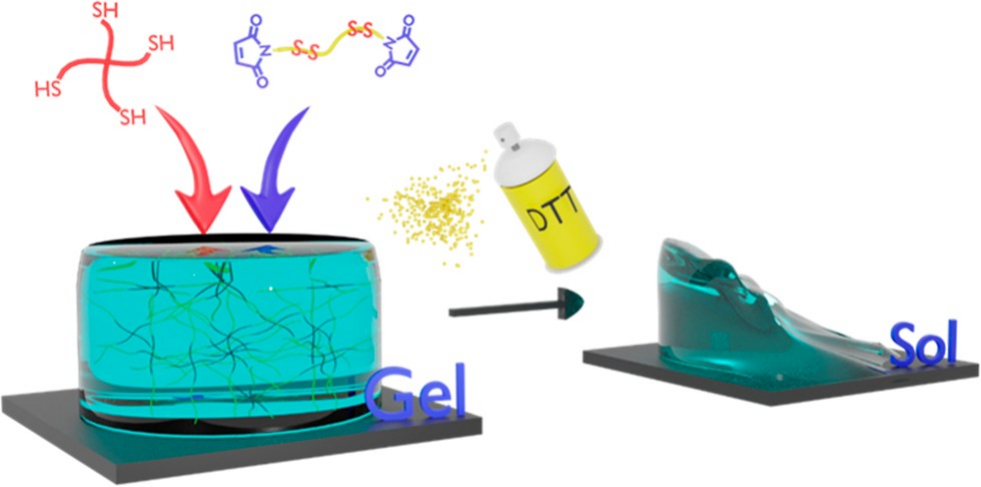

Fast-forming yet easily dissolvable hydrogels (HGs) have potential applications in wound healing, burn incidences, and delivery of therapeutic agents. Herein, a combination of a thiol–maleimide conjugation and thiol–disulfide exchange reaction is employed to fabricate fast-forming HGs which rapidly dissolve upon exposure to dithiothreitol (DTT), a nontoxic thiol-containing hydrophilic molecule. In particular, maleimide disulfide-terminated telechelic linear poly(ethylene glycol) (PEG) polymer and PEG-based tetrathiol macromonomers are employed as gel precursors, which upon mixing yield HGs within a minute. The selectivity of the thiol–maleimide conjugation in the presence of a disulfide linkage was established through ^1^H NMR spectroscopy and Ellman’s test. Rapid degradation of HGs in the presence of thiol-containing solution was evident from the reduction in storage modulus. HGs encapsulated with fluorescent dye-labeled dextran polymers and bovine serum albumin were fabricated, and their cargo release was investigated under passive and active conditions upon exposure to DTT. One can envision that the rapid gelation and fast on-demand dissolution under relatively benign conditions would make these polymeric materials attractive for a range of biomedical applications.

## Introduction

An ever-increasing utilization of hydrogels (HGs) as an attractive platform for biomedical applications such as wound healing,^1−6^ combating burn incidences,^7,8^ and local delivery of therapeutic agents ranging from small molecules to biological macromolecules such as proteins and antibodies^9−11^ has been noticeable in recent years. The choice of polymeric materials and the connectivity between the polymer chains tuned through the architecture of macromolecular building blocks or interchain crosslinking junctions enable the modulation of a wide range of properties in these materials. The HG matrix can provide one or more desirable attributes such as eliciting therapeutic agents in a localized manner at the site of disease, protecting the therapeutic agent against long-term degradation, acting as a depot for slow release, and thus eliminating frequent drug administration. The material can also act as a sponge for soaking up exudes from the wound or the site of infection. While bandages bring desirable attributes to take care of the wound/disease site, one of the issues that compromises patient comfort is the removal of bandages during routine clean-up and replacement with a new bandage.^12,13^ In light of this, the development of HG-based dissolving bandages has been investigated in recent years.^14−16^ To address this issue, installation of crosslinking junctions that can be ruptured on-demand through the application of specific stimuli such as light^17,18^ or reducing agents^19−21^ has been evaluated as a viable pragmatic approach. In recent years, HGs have also played a vital role in developing injectable formulations.^22,23^ While the HG matrix provides a suitable environment for the therapeutic agent, localized release at the disease site circumvents several problems conventional oral or intravenous administration encounters.^24,25^

HG-based materials that can be obtained through rapid gelation to afford robust crosslinked materials offer several advantages and thus would render them pragmatic for adaptation for real-life applications. In this regard, various “click” reactions have been adapted to engineer fast-forming HGs.^26,27^ For example, strain-promoted azide–alkyne cycloaddition,^28−30^ Diels–Alder reaction,^31,32^ and oxime-based click chemistry^33,34^ have been used to fabricate injectable HG systems. Among various efficient chemical transformations available to this end, the thiol–maleimide Michael addition reaction has been extensively investigated, mainly due to its rapid gelation kinetics under mild and reagent-free conditions.^35−40^ The reaction usually proceeds in the aqueous environment without any additional reagents and catalysts, and the relatively high degree of stability of this linkage in the biological milieu provides reasons for its widespread utilization. The thiol–maleimide linkage is considerably robust in nature and only upon exposure to excess competitive thiol undergoes a relatively slow degradation.^41^ In this regard, the disulfide bond is unique for its rapid cleavage upon exposure to moderate amounts of thiol, an attribute that has been widely exploited in the fabrication of stimuli-responsive polymeric materials.^42−53^ The advantage of the approach outlined here is that the complementary reactive groups used allow the formation of a disulfide-containing near-well-defined network structure. During gelation, there is no small-molecule byproduct formation; thus, gels can be used as prepared. It can be envisioned that the design of a system that embodies the rapid conjugation kinetics of the thiol–maleimide chemistry and the efficient bond scission of disulfides upon exposure to reducing conditions bear the potential to afford fast-forming crosslinked HGs with on-demand fast dissolution.

**Scheme 1:**
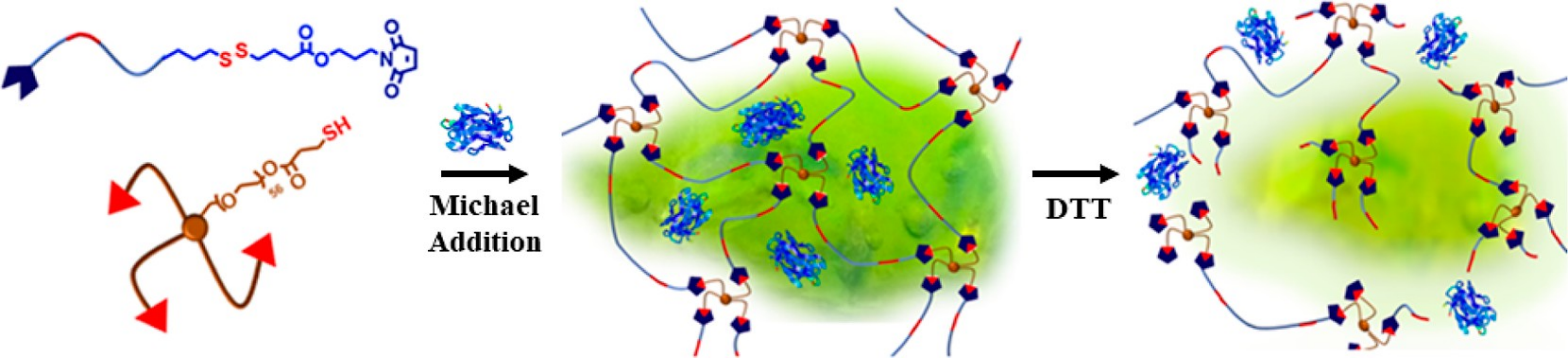
Schematic Illustration of Fabrication and Protein Encapsulation and Release from Fast-Forming Dissolvable HGs

In this study, we report the fabrication of a new class of fast-forming redox-responsive HG systems. In particular, HGs were synthesized using the Michael-type conjugation reaction between thiol-terminated tetra-arm poly(ethylene glycol) (PEG) and disulfide-containing maleimide-terminated PEG polymers (Scheme 1). The thus-obtained HGs were evaluated as platforms for sustained release of large macromolecules and proteins. Release of fluorescent dye-labeled dextran polymers and bovine serum albumin (BSA) was evaluated for stimuli-responsive triggered release.

## Experimental Section

### Reagents and Materials

Triethylamine and diethyl ether were purchased from Merck. 1,4-Dithio-dl-threitol (DTT) (98%) and trifluoroacetic acid were purchased from Alfa Aesar. 4-(Dimethylamino)pyridine (DMAP, >99%), 4,4′-dithiodibutyric acid (95%), 3-mercaptopropionic acid, *N*,*N*′-dicyclohexylcarbodiimide (DCC), PEG (4 kDa), toluene, fluorescein isothiocyanate–dextran (FITC-dextran, 20, 150 kDa), albumin–FITC conjugate (FITC–BSA), and *p*-toluenesulfonic acid monohydrate (99%) were purchased from Sigma-Aldrich. Four-arm PEG (10 kDa) was purchased from Creative PEGWorks. The thiol-terminated four-arm PEG^54^ and the furan-maleimide adduct containing alcohol^55^ were obtained using literature procedures.

### Instrumentation

Please see the Supporting Information for instrumentation details.

### Cell Lines

L929 mouse fibroblast cell lines from ATCC (LGC Standards, Germany) were used. For experimental details, please see the Supporting Information.

### Synthesis of Masked Maleimide Disulfide Acid

4,4′-Dithiodibutyric acid (2.16 g, 0.010 mol), furan-protected maleimide-containing alcohol (1.00 g, 0.004 mol), and DMAP (0.350 g, 0.003 mol) were added to a round-bottom flask and dissolved in anhydrous dichloromethane (40 mL). In another flask, DCC (2.00 g, 0.009 mol) was dissolved in dichloromethane (20 mL). The content of the second flask was slowly added to the ice-cold mixture in the first flask. The combined reaction mixture was stirred at room temperature for 24 h. To the reaction mixture was added dichloromethane (25 mL), and then, the precipitated dicyclohexylurea was removed by filtration, followed by washing of the organic phase with a saturated NaHCO_3_ solution. Anhydrous Na_2_SO_4_ was utilized to dry the organic layer, and it was concentrated under low pressure. Purification using column chromatography on SiO_2_ using CH_2_Cl_2_ and EtOAc (1:1) as eluents yielded the pure product (1.90 g, 80%). ^1^H NMR (CDCl_3_, δ, ppm), 6.50 (s, 2H, CH=CH), 5.26 (s, 2H, CH bridge protons), 4.02 (t, 2H, OCH_2_CH_2_), 3.57 (t, 2H, NCH_2_CH_2_), 2.85 (s, 2H bridgehead protons), 2.72 (t, 4H, SCH_2_CH_2_), 2.47 (t, 4H, COCH_2_CH_2_), 2.03 (m, 4H, SCH_2_CH_2_CH_2_), 1.94 (m, 2H, NCH_2_CH_2_CH_2_).

### Synthesis of Maleimide Disulfide-Terminated Telechelic PEG Polymer

Masked maleimide disulfide acid (0.25 g, 0.500 mmol), PEG (0.56 g, 0.140 mmol), and DMAP (0.01 g, 0.080 mmol) were added to a round-bottom flask and dissolved in dichloromethane (0.35 mL). DCC (0.12 g, 0.550 mmol) was added to another round-bottom flask and dissolved in dichloromethane (0.20 mL). The first solution was cooled down, and the second solution was added to the ice-cold mixture slowly, and the reaction was stirred at room temperature for 24 h. Dichloromethane (5 mL) was added to the reaction mixture, and the precipitated dicyclohexylurea was removed by filtration, and the concentrated solution was precipitated in ether. ^1^H NMR (CDCl_3_, δ, ppm), 6.50 (s, 4H, CH=CH), 5.26 (s, 4H, CH bridgehead protons), 4.25 (t, 4H, OCH_2_CH_2_O), 4.02 (t, 4H, OCH_2_CH_2_), 3.40–3.80 (broad s, 360 H), 2.85 (s, 4H bridge protons), 2.72 (t, 8H, SCH_2_CH_2_), 2.47 (t, 8H, COCH_2_CH_2_), 2.03 (m, 8H, SCH_2_CH_2_CH_2_), 1.94 (m, 4H, NCH_2_CH_2_CH_2_). The furan protection was removed by dissolving the above compound (0.65 g) in toluene (20 mL). The solution was refluxed for 10 h. The concentrated solution was purified by precipitation with diethyl ether to yield the PEG-based bismaleimide crosslinker (92% yield). ^1^H NMR (CDCl_3_, δ, ppm), 6.70 (s, 4H, CH=CH), 4.25 (t, 4H, OCH_2_CH_2_O), 4.02 (t, 4H, OCH_2_CH_2_), 3.40–3.80 (broad s, 360 H), 2.72 (t, 8H, SCH_2_CH_2_), 2.47 (t, 8H, COCH_2_CH_2_), 2.03 (m, 8H, SCH_2_CH_2_CH_2_), 1.94 (m, 4H, NCH_2_CH_2_CH_2_).

### Selectivity in Thiol–Maleimide and Thiol–Disulfide Exchange Reactions

For a 1:1 M ratio of the maleimide/thiol reaction, maleimide disulfide-terminated PEG polymer (10 mg) and 2-mercaptoethanol (0.30 mg) were dissolved in phosphate-buffered saline (PBS) (1× 100 μL) and stirred at 200 rpm for 30 min at 37 °C. The reaction solution was then freeze-dried and redissolved in CH_2_Cl_2_. The solution was precipitated in 1.5 mL of diethyl ether and centrifuged at 17,000 rpm for 5 min to remove the polymeric component. The supernatant was passed through a short silica gel plug using methanol as an eluent. The solution was then evaporated and redissolved in acetonitrile (1 mL) and analyzed by liquid chromatography–mass spectrometry (LC–MS). The same procedure was also applied for the reaction where a maleimide/thiol ratio of 1:4 was utilized.

### Preparation and Characterization of the HGs

HGs were prepared by rapidly mixing the two polymer solutions. Briefly, maleimide disulfide-terminated telechelic PEG polymer (10 mg) and thiol-terminated four-arm PEG polymer (10 mg) were separately dissolved in 50 μL of PBS solutions. Solutions were sonicated for 1 min, and then, one solution was added into the other. The HG was formed upon mixing the two polymer solutions. To ensure high interchain coupling, the mixture was incubated at 37 °C for 30 min. For loading of FITC-labeled protein and dextran polymers, the maleimide-containing HG precursor was dissolved in FITC-labeled macromolecule or protein-containing PBS solution, and HGs were prepared with the process described above. Dye-labeled polymers and proteins were encapsulated in a quantitative manner. Lyophilized HG disks (ca. 80 mg) were dipped in deionized water at 37 °C. The HG samples were periodically weighed after removing adherent water from their surface using a tissue paper. The water uptake was recorded until no additional increase in weight was observable. Swelling ratio percentages were calculated using (*M*_s_ – *M*_dry_)/*M*_dry_ × 100, where *M*_dry_ and *M*_s_ refer to the weight of dry and swollen HGs, respectively. The linear viscoelastic limit regime (LVR) and degradation profiles of HGs were recorded using an Anton Paar MCR 302 rheometer. A 15 mm diameter plate was utilized for the tests. The LVR of HGs was determined using the strain sweep test. Nonlinear strain regions indicate breakdown in HGs and were regimes that were out of the LVR range. The frequency sweep test allows us to ascertain the lower frequency limit where gel-like behavior can be observed. The frequency sweep test with a suitable strain value enabled determination of the frequency range for doing time sweep tests. A time sweep test with a strain of 1% and an angular frequency of 10 rad/s was employed to follow the degradation process. A time sweep test was performed with a swollen HG between preheated rheometer plates and DTT or glutathione solution around the HG. To minimize any effect of solvent loss due to vaporization, a closed chamber was used for degradation experiments.

### Degradation of the HG and FITC-Labeled Protein and Dextran Release

Two pieces of the disk-shaped HG were kept at 37 °C in a thermal shaker (200 rpm) in 1 mL of PBS solution and 200 mM DTT containing 1 mL of PBS solution. The degradation process of bulk HGs for 10 min was recorded using a digital camera. FITC–BSA and FITC-dextran (150 and 20 kDa) were encapsulated in HGs as described above. After gelation, HG disks (1 cm diameter, 2 mm thick) were placed in an Eppendorf tube with 1 mL of PBS and DTT (10 mM DTT in PBS). The amount of biomolecule and dextran polymer release in the supernatant was obtained using a UV–vis spectrophotometer.

### Cytotoxicity Experiments

Cytotoxicity of the HGs was investigated via a CCK-8 viability assay on L929 mouse fibroblast cells. Cells (6000 cells/well) were seeded on a 96-well plate in quadruplicate with 100 μL of the culture medium and incubated at 37 °C overnight to grow and adhere completely. Cells were treated with gels (1.0, 0.5, and 0.1 mg) for 48 h. After the incubation time, gels were removed, and cells were treated with a 10% CCK-8 solution for 3 h, and the absorbance values at 450 nm were measured using a microplate reader. Viability results were obtained on GraphPad prism software in the nonlinear regression mode.

### Live/Dead Cell Viability Assay

L929 cells were seeded in a 12-well plate (200,000 cells/well) with Dulbecco’s modified Eagle’s medium (low) and incubated at 37 °C overnight to grow and adhere. After the incubation process, the medium was removed, and cells were treated with varying amounts of the HGs for 24 h. Thereafter, HGs were removed, and cells were rinsed twice with PBS. Finally, cells were stained according to the protocol of the live/dead assay kit (Sigma, 04511-1KT-F). The cells were stained with PBS solution containing 10 μL of calcein-AM and then with 5 μL of propidium iodide (PI) for 30 min at 37 °C. After removing the solution and washing with PBS, the cells were imaged using a fluorescence microscope (Zeiss Observer A1 equipped with AxioCam MRc5) and AxioVision software. The live cells exhibited green fluorescence due to calcein-AM, and dead cells showed red fluorescence due to PI.

## Results and Discussion

### Synthesis and Characterization of Maleimide Disulfide-Terminated Telechelic PEG Polymer

**Figure 1:**
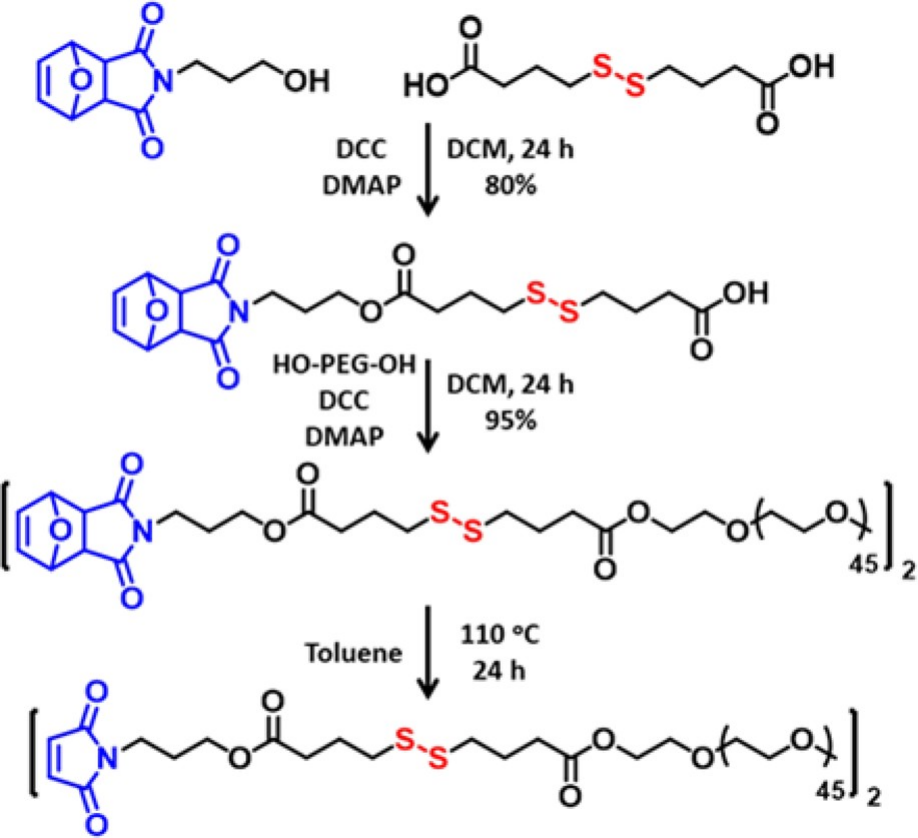
Synthesis of the maleimide disulfide-terminated telechelic PEG polymer.

The telechelic PEG-based polymeric component containing maleimide functional groups linked through a disulfide linker was derived from a readily available furan-protected maleimide-containing alcohol (Figure 1). To yield the desired chain-end functionality, the furan-protected maleimide-containing alcohol fragment was linked to 4,4′-dithiodibutyric acid through esterification. The composition and purity of the synthesized molecule were confirmed using ^1^H and ^13^C NMR spectroscopy, Fourier transform infrared (FTIR) spectroscopy, and LC–MS analysis (Figures S1–S3). The FTIR spectrum exhibited the expected carboxylic acid and carbonyl bands at around 3200 and at 1699 cm^–1^, respectively (Figure S1). The ^1^H NMR spectrum exhibited the presence of proton resonances at 6.50 and 5.26 ppm belonging to protons on the bicyclic moiety and at 2.72 ppm belonging to protons adjacent to the disulfide unit, confirming the structure (Figure S3A). In the ^13^C NMR spectrum, the resonances at 176.3 and 172.9 ppm belonging to imide and ester carbonyls and at 178.0 ppm belonging to carbon on the carboxylic acid were evident (Figure S3B). The esterification reaction was used for coupling this fragment with a linear PEG polymer to yield the masked maleimide disulfide-terminated telechelic PEG polymer as a HG precursor (Figure 1). The composition and purity of the functionalized linear PEG polymer were confirmed using ^1^H NMR (Figure S4) and ^13^C NMR (Figure S5) spectroscopy. The proton resonances at 6.50 and 4.25 ppm belonging to the vinylic protons on the bicyclic structure and ester protons next to the hydroxyl unit of the precursor PEG, respectively, indicated successful chain-end modification.

**Figure 2:**
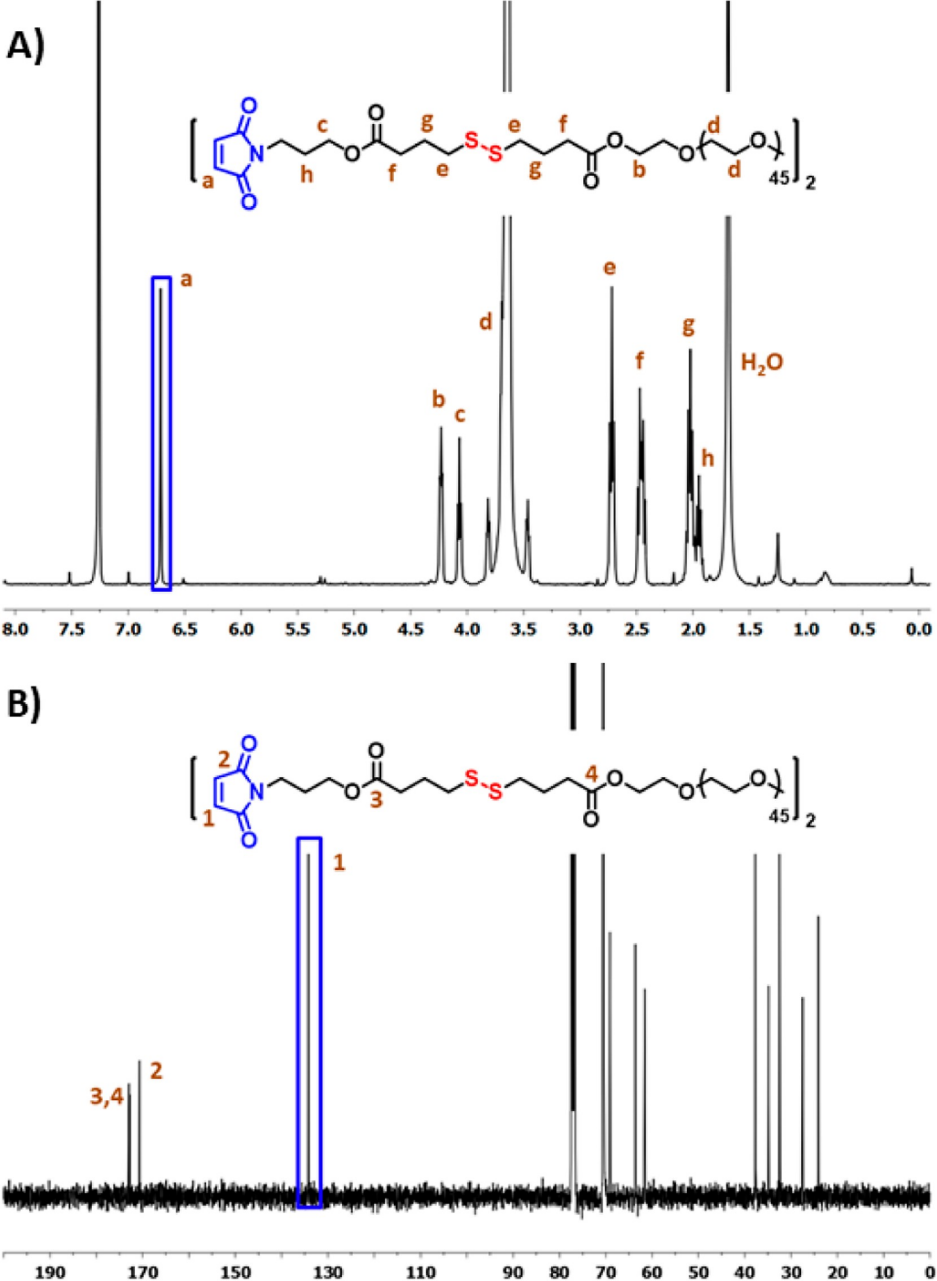
(A) ^1^H and (B) ^13^C NMR spectra of maleimide disulfide-terminated PEG polymer.

Removal of the furan protection group to obtain the thiol-reactive maleimide form was accomplished via the retro Diels–Alder reaction by refluxing in anhydrous toluene. The composition and purity of this HG precursor were also confirmed using ^1^H and ^13^C NMR spectroscopy. The presence of a proton resonance at 6.70 ppm belonging to the vinylic protons on the maleimide ring and other expected proton resonances confirmed the structure (Figure 2A). The carbon resonance at 134.2 ppm belonging to the vinylic carbon on the maleimide ring and at 170.6 ppm belonging to the carbonyl group on the maleimide ring also confirmed the presence of this reactive group (Figure 2B). The successful synthesis of the maleimide disulfide-terminated telechelic PEG polymer was also confirmed using FTIR. After synthesizing masked maleimide disulfide-terminated PEG, new carbonyl peaks of ester bonds and imide groups appeared at 1731 and 1699 cm^–1^, respectively (Figure S6). After the retro-Diels–Alder reaction, carbonyl peaks of imide groups shifted to 1704 cm^–1^, and a new peak appeared at 696 cm^–1^ corresponding to the C–H vibration of the maleimide moiety.^56^

### Orthogonality of the Thiol–Maleimide and Thiol–Disulfide Exchange Chemistry

**Figure 3:**
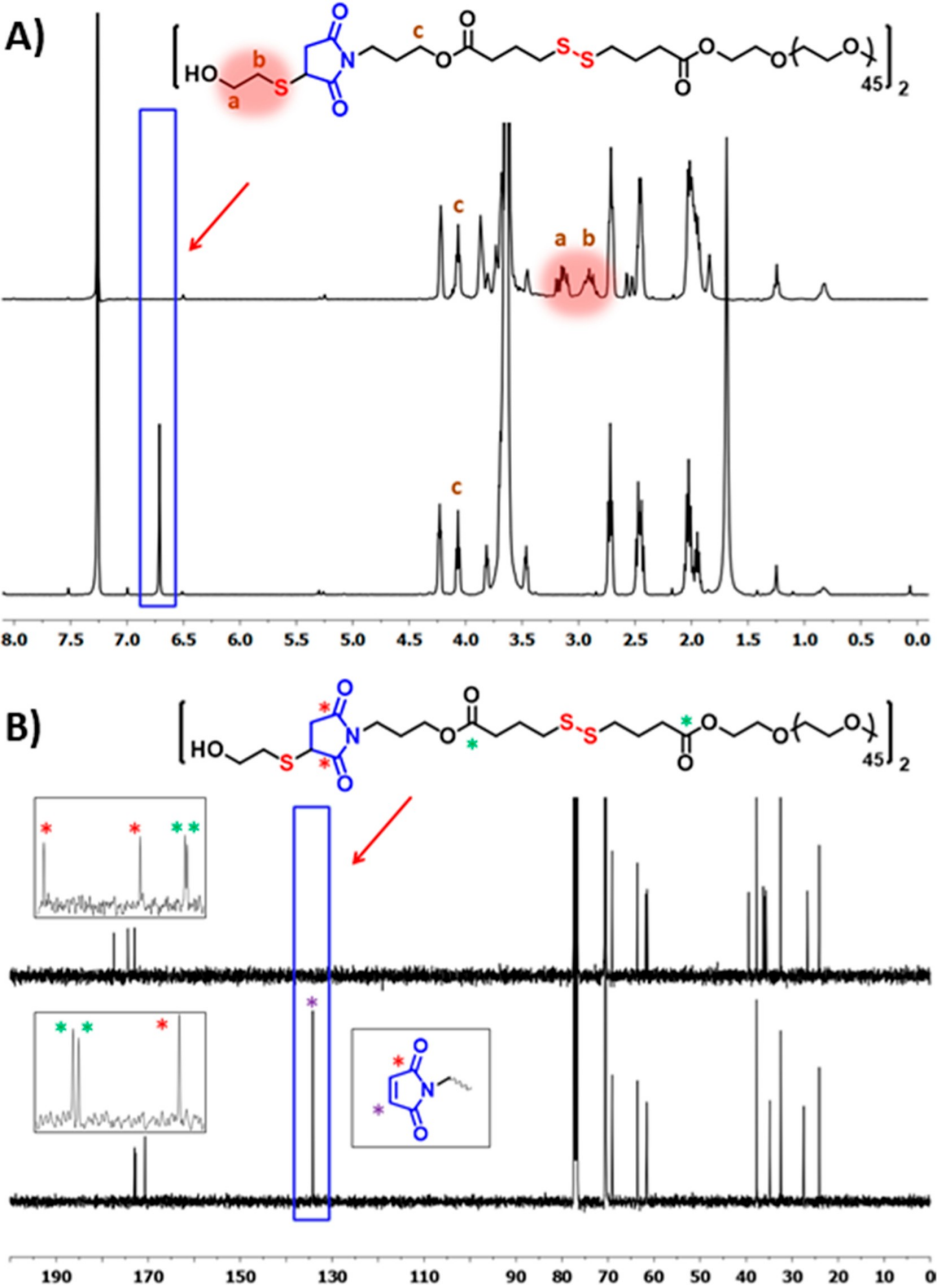
Comparison of (A) ^1^H and (B) ^13^C NMR spectra of the PEG bismaleimide polymer and thiol-conjugated bismaleimide polymers.

At this point, it was important to confirm that the HG precursors that contain two different thiol-reactive bonds: the maleimide and the disulfide groups will have a much higher propensity to undergo the thiol–maleimide conjugate addition compared to the thiol–disulfide exchange reaction. The selectivity of thiol addition was investigated by treatment of the maleimide disulfide-terminated telechelic PEG polymer with the stoichiometric equivalent of 2-mercaptoethanol. The ^1^H NMR analysis of obtained polymer revealed that the proton resonance at 6.70 ppm belonging to the vinylic proton on the maleimide ring had completely disappeared, while new proton resonance from the 2-mercaptoethanol fragment appeared (Figure 3A). The ^13^C NMR analysis demonstrated that the vinylic carbon resonance at 134.2 had completely vanished. The resonance belonging to carbonyls on the maleimide ring had also shifted to 174.5 and 177.5 as two different peaks due to the thiol–maleimide conjugation (Figure 3B). As expected, there was minimal change in the size-exclusion chromatography of the bismaleimide-containing polymer after thiol-conjugation (Figure S7). Furthermore, the solution of the reaction of the maleimide disulfide-terminated PEG with the stoichiometric equivalent of 2-mercaptoethanol was also analyzed using LC–MS, and no side product due to disulfide cleavage was observed (Figure S8A). On the other hand, using an excess (4 equiv) of 2-mercaptoethanol led to the formation of products from disulfide bond cleavage (Figure S8B). Hence, it was inferred that for these constructs, the thiol groups preferentially undergo a Michael addition reaction with electron-deficient conjugated alkenes compared to thiol–disulfide exchange reactions with disulfide linkages.

### Preparation and Characterization of HGs

**Figure 4:**
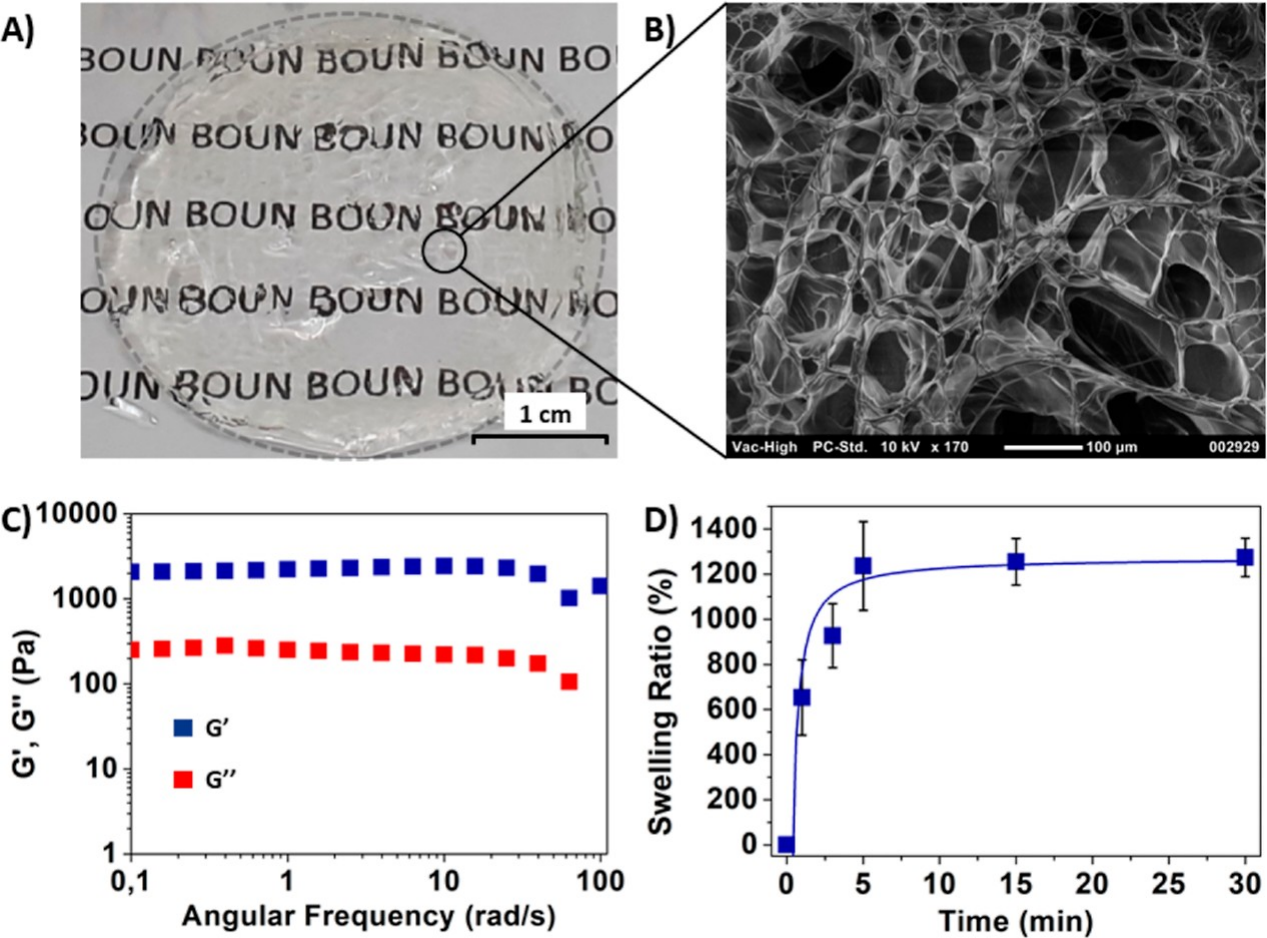
(A) Photographic image of a wet HG sample, (B) SEM image of a dry HG sample (scale bar: 100 μm), (C) frequency sweep test, and (D) water uptake swelling profile.

For the preparation of HGs, the maleimide disulfide-terminated telechelic PEG polymer was mixed with a tetra-arm thiol-containing PEG polymer in PBS solution at 37 °C, with an equimolar stoichiometry of the thiol and maleimide functional groups. Clear and transparent HGs formed within a minute of mixing the polymers, with near-quantitative conversions (Figure 4A). The morphology of the obtained HG was investigated using scanning electron microscopy (SEM), and a highly porous structure was observed (Figure 4B). Lack of the appearance of any characteristic UV–vis absorbance peak upon treatment of HGs with Ellman’s reagent suggested that no significant amount of free thiol groups was present in the obtained HGs, as opposed to the peak observed at 411 cm^–1^ for the tetra-thiol polymeric precursor (Figure S9). The gelation product was analyzed using FTIR spectroscopy, where it was observed that the characteristic peak of imide carbonyl groups shifted from 1704 to 1706 cm^–1^ and appeared as a smaller peak as well as the absorbance of a maleimide moiety at 696 cm^–1^ disappeared (Figure S10), which are in accordance with the results of a previous work related to thiol-maleimide additions.^56^ To investigate the stability and degradation profile of the HG, rheological behaviors of a water-swollen HG were studied via a strain sweep test and frequency sweep test. A strain sweep test was performed to investigate the linear viscoelastic limits of HGs. When 0.01–100% strain was applied on the HG, a linear viscoelastic behavior was observed up to 4% strain under 10 rad/s frequency, which indicates a lack of structural breakdowns (Figure S11). The response of HGs to scaled frequency was measured by the frequency sweep test from 0.01 to 100 rad/s with a constant strain of 1% (Figure 4C). As expected for gels prepared with hydrophilic PEG polymers, HGs exhibited a rapid high water uptake within a few minutes (Figure 4D).

### Degradation of HGs

**Figure 5:**
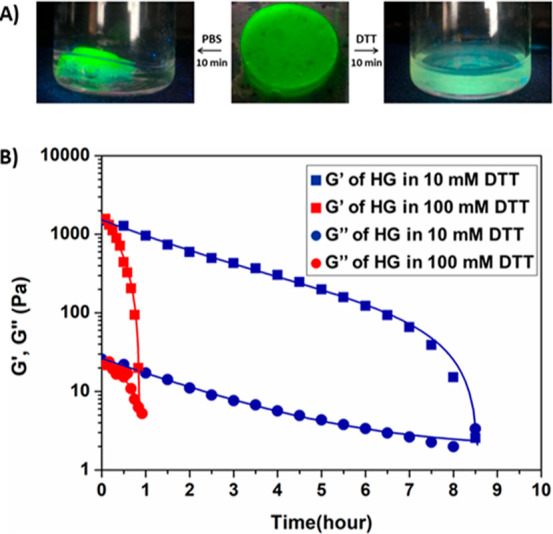
(A) Degradation behavior of the disulfide-containing HG in PBS and DTT (200 mM) solutions and (B) rheological analysis of the degradation of the HG in DTT solutions (10 and 100 mM).

To monitor the degradation behavior in reductive and nonreductive environments, HGs loaded with a fluorescent dye-labeled protein, namely, FITC–BSA, were prepared. To investigate the effect of FITC–BSA loading on the rheological properties of HG, strain and frequency sweep tests were undertaken, and no significant change compared to the protein-free HGs was observed (Figure S12). The obtained FITC–BSA-loaded swollen disk-shaped HG was cut into two pieces, and one of these pieces was immersed in PBS solution, while the other one was immersed in DTT solution (200 mM) at 37 °C (Figure 5A). As expected, while there was no visible degradation for the HG immersed in PBS solution over a prolonged period, the HG immersed in DTT solution degraded completely, and the solution became fluorescent within 10 min. Hence, as expected from the molecular design of these materials, they undergo rapid gelation to provide stable HGs as well as undergo rapid dissolution in the presence of a thiol-based reducing agent (see Video S1).

Degradation of HGs was also probed using the time sweep test using a rheometer. For the time sweep test, the HG sample was placed between parallel plates, and the chamber around the HG was filled with either PBS or DTT solution. It was observed that the elastic modulus of HG remained constant when the chamber was filled with PBS solution (Figure S13). However, for the HG that was incubated in the DTT solution, a drastic decrease in elastic modulus was observed. While in a 10 mM DTT solution, it takes 8 h, in a 100 mM DTT solution, it takes only 40 min for complete degradation of the HG (Figure 5B). As expected, glutathione works relatively slower than DTT in terms of disulfide bond cleavage. It was observed that complete degradation took 30 h when the HG was placed in a 10 mM glutathione solution (Figure S6). This difference in disulfide bond scission is in accordance with the higher reducing activity of DTT compared to that of GSH.^57^

### Macromolecular Release from HGs

**Figure 6:**
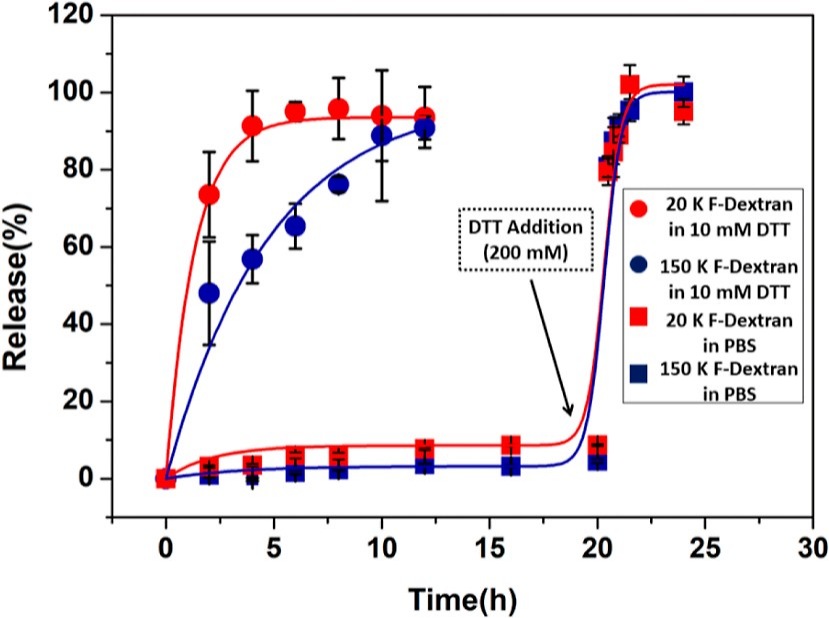
Release profiles of 150 and 20 kDa FITC-dextran (F-Dextran) from HGs in DTT and PBS solutions.

Obtained HGs were utilized to study the release of macromolecules such as polymers and proteins, which are often employed as macromolecular therapeutic agents. First, FITC-dextran polymers (20 and 150 kDa) were used to probe their release from the HG network. Two different molecular weights of FITC-dextran were used to ascertain the effect of molecular weight on release. Briefly, the FITC-dextran polymer and maleimide disulfide-terminated PEG polymer were dissolved in PBS. This solution was quickly mixed with a thiol-terminated PEG polymer dissolved in PBS. A solid and freestanding transparent HG was obtained. Thereafter, HGs were immersed in DTT (10 mM) and PBS solutions to investigate the release of the FITC-dextran polymer. It was noticeable that the cumulative release of FITC-dextran was considerably higher in DTT solution than in PBS (Figure 6). As expected, relatively less release was observed for the 150 kDa FITC-dextran polymer, while more dextran release was observed for the lower molecular weight 20 kDa FITC-dextran. To demonstrate the on-demand release, HGs were treated with a concentrated DTT solution. For this purpose, the release solution of HGs was exchanged with a 200 mM DTT solution after 20 h. Under this highly reducing environment, HGs degraded rapidly and released all of the encapsulated FITC-labeled polymers (Figure 6).

**Figure 7:**
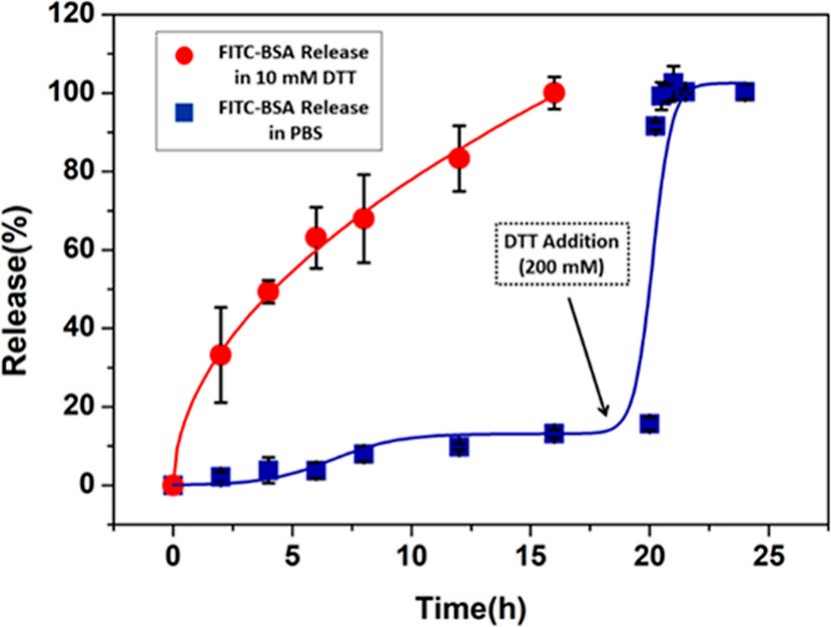
Release profile of FITC–BSA protein from HGs immersed in reducing and nonreducing environments.

Additionally, as a model protein, FITC–BSA was utilized to investigate the release profile of biomacromolecules. Using the above-mentioned protocol, HGs were prepared to obtain a freestanding HG loaded with FITC–BSA. Obtained HGs were immersed in DTT (10 mM) and PBS solutions. As expected, the cumulative release of FITC–BSA was substantially higher in the DTT solution than in the PBS solution (Figure 7). While the release in PBS solution was slow, the addition of a 200 mM DTT solution initiated the forced release, whereby the HG degraded completely and released all of the encapsulated protein within a few minutes. It should be noted that FITC–BSA was used here as a model example to demonstrate that tunable release of large biomacromolecules from such gels is possible. One can envision that the concept can be extended to release of other large macromolecules such as oligonucleotide sequences, as well as drug-conjugated polymers encapsulated within such HGs, but may be limited to biomolecules which do not possess susceptible disulfide units whose cleavage under reducing conditions may lead to loss of activity, unless they are able to refold to their original structure after release from the reducing environment.

**Figure 8:**
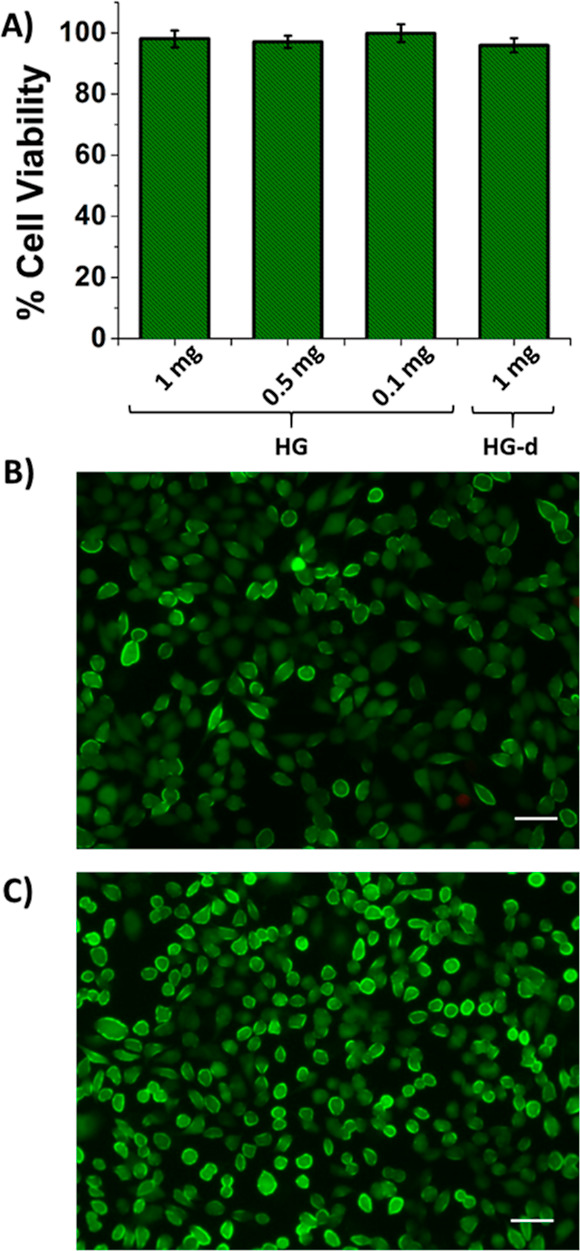
(A) Cell viability of L929 fibroblasts upon treatment with varying amount of HGs and with the degradation product of the HG (HG-d). Fluorescence microscopy images of the live/dead cell viability assay upon treatment with the (B) HG and (C) with the degradation product of hydrogel (scale bar in images: 50 μm).

The in vitro cytotoxicity of the HG was evaluated on a L929 fibroblast cell line using the CCK-8 cell viability assay and live/dead assay. The results CCK-8 (Figure 8a) and live/dead cell viability assays (Figure 8b) showed no significant cytotoxicity when cells were treated with varying amounts of HGs. Furthermore, to evaluate the cytotoxicity of degradation products (HG-d), a HG sample was incubated in DTT solution (10 mM) for 6 h at 37 °C. The cytotoxicity of the supernatant was evaluated on the L929 fibroblast cell line using the CCK-8 cell viability assay (Figure 8a) and live/dead cell viability assay (Figure 8c). No significant cytotoxicity was observed in either of the experiments, which suggests the benign nature of these materials.

## Conclusions

In conclusion, redox-responsive HGs were fabricated using tetra-arm PEG thiol and maleimide disulfide-terminated telechelic linear PEG polymers. Efficient crosslinking between these polymers was accomplished through the Michael addition reaction without the need for any additional catalyst. Fluorescent dye-labeled macromolecules and proteins could be efficiently encapsulated under mild conditions. While a slow release of these macromolecules was observed in a nonreducing environment, exposure to a reducing milieu accelerated the process. Notably, the entire HG construct can be rapidly dissolved upon the addition of a concentrated DTT solution. One can envision that such control over the release of macromolecular agents and on-demand HG dissolution would be attractive for adapting these materials for different biomedical applications.

## Supporting Information Available

^1^H NMR, ^13^C NMR, and LC–MS data; SEC plots of polymeric precursors; and rheological data of HGs (PDF)
Formation and dissolution of a HG (AVI)

## Supplementary Material

bm2c00209_si_001.pdf

bm2c00209_si_002.avi
