# Supplementary material for: Fast-Forming Dissolvable Redox-Responsive Hydrogels: Exploiting the Orthogonality of Thiol–Maleimide and Thiol–Disulfide Exchange Chemistry
Source: Biomacromolecules. 2022 Jun 13;23(9):3525–34. doi: 10.1021/acs.biomac.2c00209 (PMC9472223; doi:10.1021/acs.biomac.2c00209)
Supplement: Supplementary file 1 — bm2c00209_si_001.pdf [file bm2c00209_si_001.pdf]

Fast-Forming Dissolvable Redox-Responsive Hydrogels: Exploiting Orthogonality of the Thiol-Maleimide and Thiol-Disulfide Exchange Chemistry

Ismail Altinbasak,[†] *Salli Kocak,*[†] *Rana Sanyal,*[†,‡] *and Amitav Sanyal,**[†,‡]

†Department of Chemistry and ‡Center for Life Sciences and Technologies,

Bogazici University, Bebek, Istanbul 34342, Turkey

LIST OF FIGURES

Figure S1. FTIR spectrum of masked maleimide-disulfide acid…………………..……………...4

Figure S2. LCMS characterization of the masked maleimide-disulfide acid………………..…....4

Figure S3. A) ^{1}H NMR and B) ^{13}C NMR spectra of the masked maleimide-disulfide acid...……5

Figure S4. ^{1}H NMR spectrum of the masked maleimide-disulfide terminated telechelic PEG polymer…………………………………………………………………………………….5

Figure S5. ^{13}C NMR spectrum of the masked maleimide-disulfide terminated telechelic PEG polymer…………………………………………………………………………………….6

Figure S6. FTIR spectra of PEG, masked maleimide-disulfide terminated PEG, and maleimide-disulfide terminated PEG……………………………………………..………………………6

Figure S7. SEC plots of PEG polymers before and after addition of 2-mercaptoethanol…………..7

Figure S8. LCMS characterization of the solution of the reaction between maleimide-disulfide terminated PEG with (A) the stoichiometric equivalent of 2-mercaptoethanol and (B) four equivalent of 2-mercaptoethanol……………………………………………………..…….8

Figure S9. UV-VIS spectra of Ellman's reagent solution incubated with hydrogel………………9

Figure S10. FTIR spectra of thiol-terminated four arm PEG, maleimide-disulfide terminated PEG, and hydrogel……………………………………………………………………..……………9

Figure S11. Strain sweep test of the hydrogel…………………………………………....….10

Figure S12. A) Frequency sweep test and B) strain sweep test of the FITC-BSA loaded hydrogel.10

Figure S13. Time sweep test of hydrogel in presence and absence of glutathione (GSH)………...11

Instrumentation. Ultra-pure water was obtained via MilliQ Water Purification System (Merck Millipore, USA). FTIR spectroscopy measurements were done on a Thermo Scientific Nicolet 380 FTIR spectrometer. NMR analysis was carried out using a 400 MHz Bruker spectrometer. Molecular weights of the polymers were monitored using a gel permeation chromatography using a PSS-SDV (gram linear, length/ID 8 × 300 mm, 10 µm particle size) column calibrated with poly(methyl methacrylate) standards (1−175 kDa) using a refractive index detector. Dimethylacetamide with 0.05% lithium bromide was used as an eluent at a flow rate of 1.0 mL/min at 30 °C. The surface morphology of hydrogels was examined using scanning electron microscopy (SEM) (JEOL NeoScope JCM-500, with an accelerating voltage of 10 kV). The mechanical properties of hydrogels were analyzed using a rheometer (Anton PAAR MCR 302). UV-Vis spectrophotometer measurements were carried out using Thermo Scientific Nanodrop 2000 spectrophotometer and a Varian Cary Eclipse, Agilent, USA). Side products of reactions were quantified by liquid chromatography-mass spectrometry (LC-MS) analysis using an LCMS-2020-mass spectrometer system (Shimadzu, Japan) equipped with a C-18, 5 µm, 150 × 4.6 mm column. The mobile phase consisted of deionized water and HPLC grade acetonitrile (ACN) using the following gradient: LC: 0–14 min, 50% ACN; 18.01 min, 95% ACN; 25 min, 95% ACN; 25.01–30 min, 50% ACN.

Cell Lines. L929 mouse fibroblast cell line was purchased from ATCC (LGC Standards, Germany) and grown according to the culture methods requirements of the manufacturer. Cells were kept in the logarithmic phase of cell growth for the duration of experiments and incubated in a humidified atmosphere of 5% CO_2 at 37 °C. Cytotoxicity experiments were done with a plate reader (Multiscan FC, Thermo Scientific, USA), and Cell Counting Kit8 (CCK-8, Fluka) was obtained from Sigma-Aldrich.

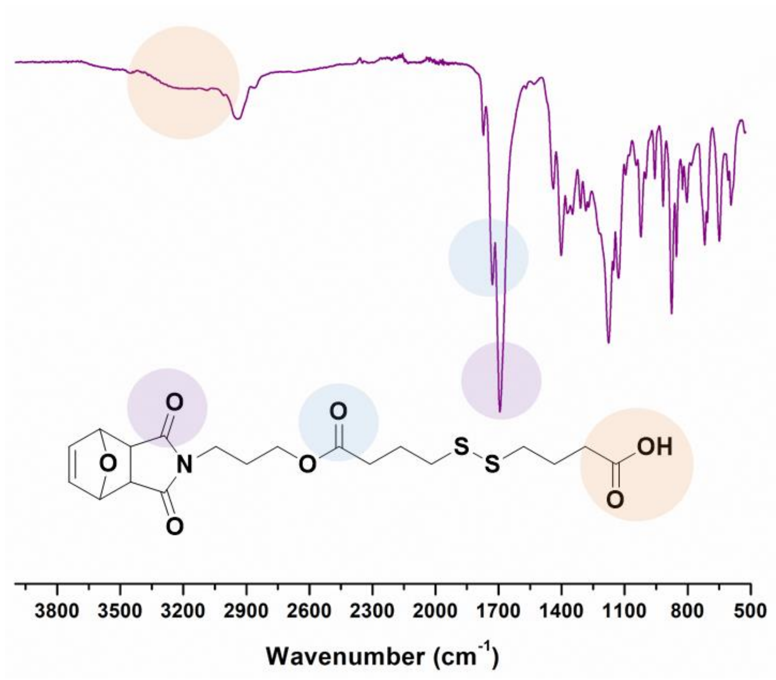

Figure S1. FTIR spectrum of masked maleimide-disulfide acid.

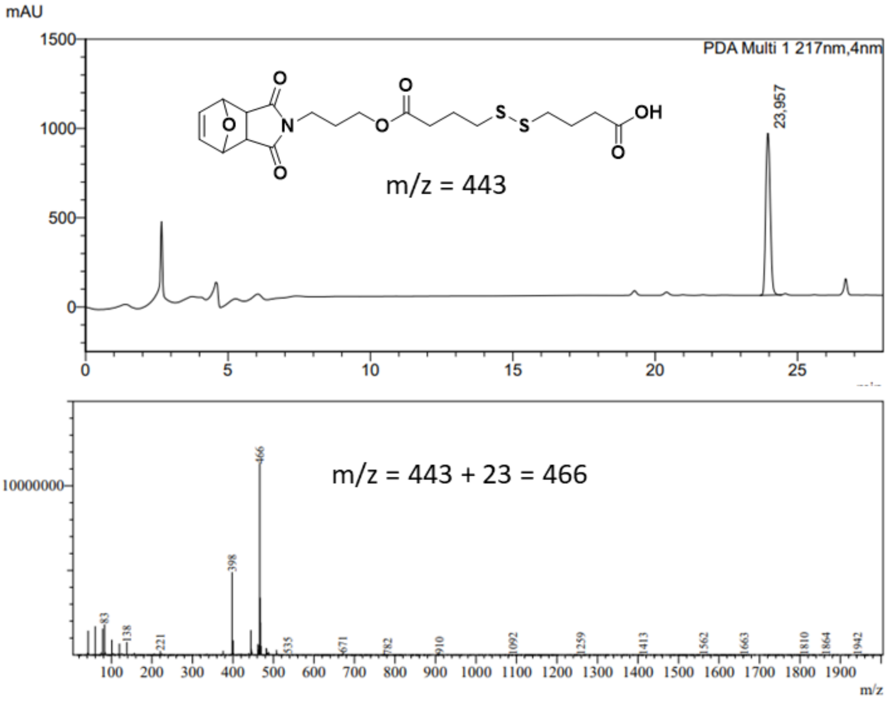

Figure S2. LCMS characterization of the masked maleimide-disulfide acid.

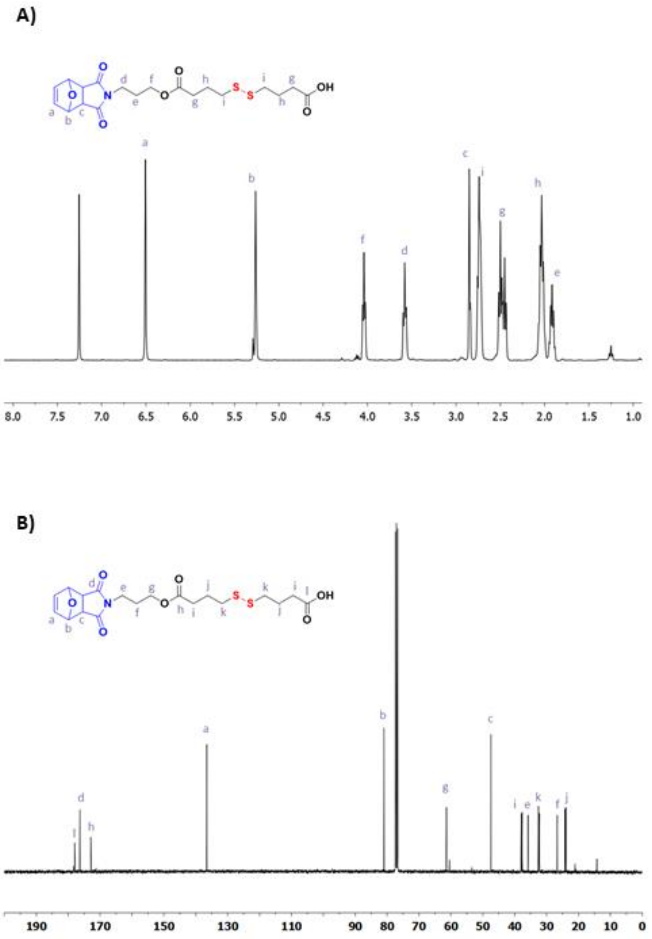

Figure S3. A) ^{1}H NMR and B) ^{13}C NMR spectra of the masked maleimide-disulfide acid.

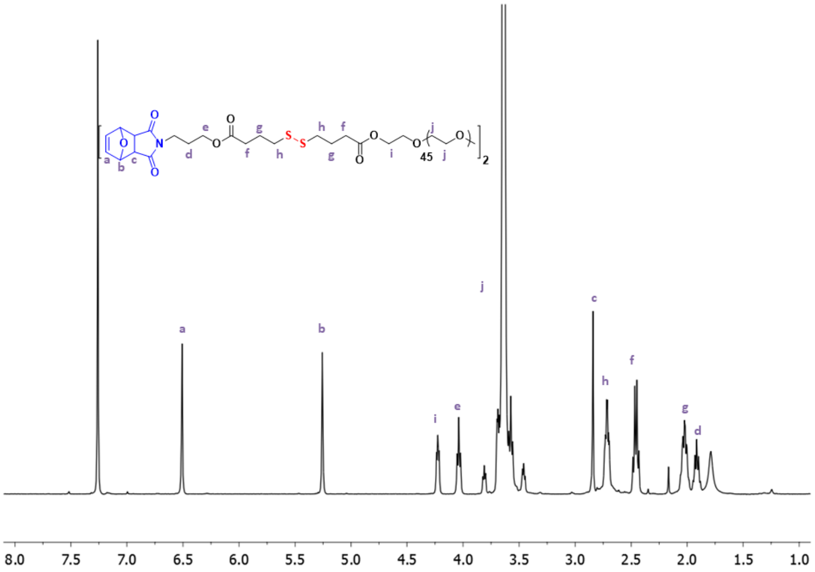

Figure S4. ^{1}H NMR spectrum of the masked maleimide-disulfide terminated telechelic PEG polymer.

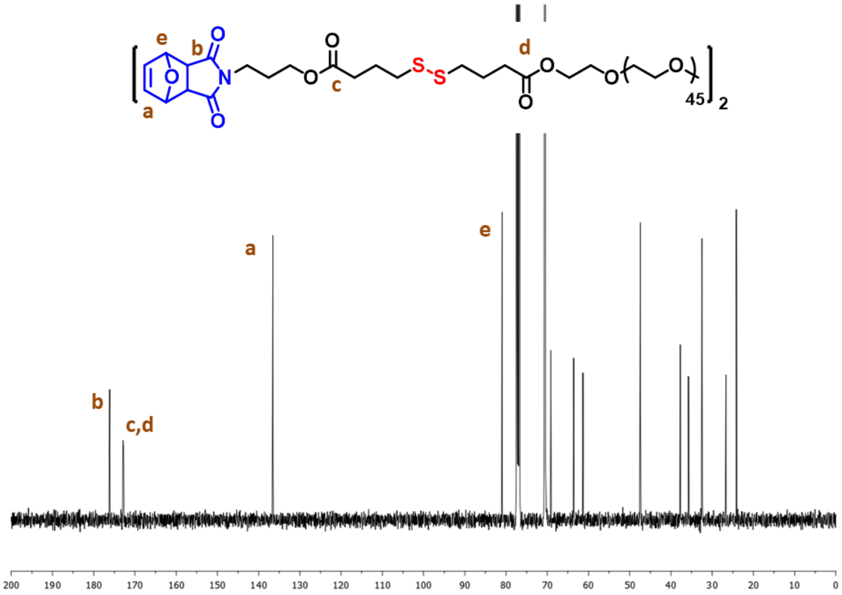

Figure S5. ^{13}C NMR spectrum of the masked maleimide-disulfide terminated telechelic PEG polymer.

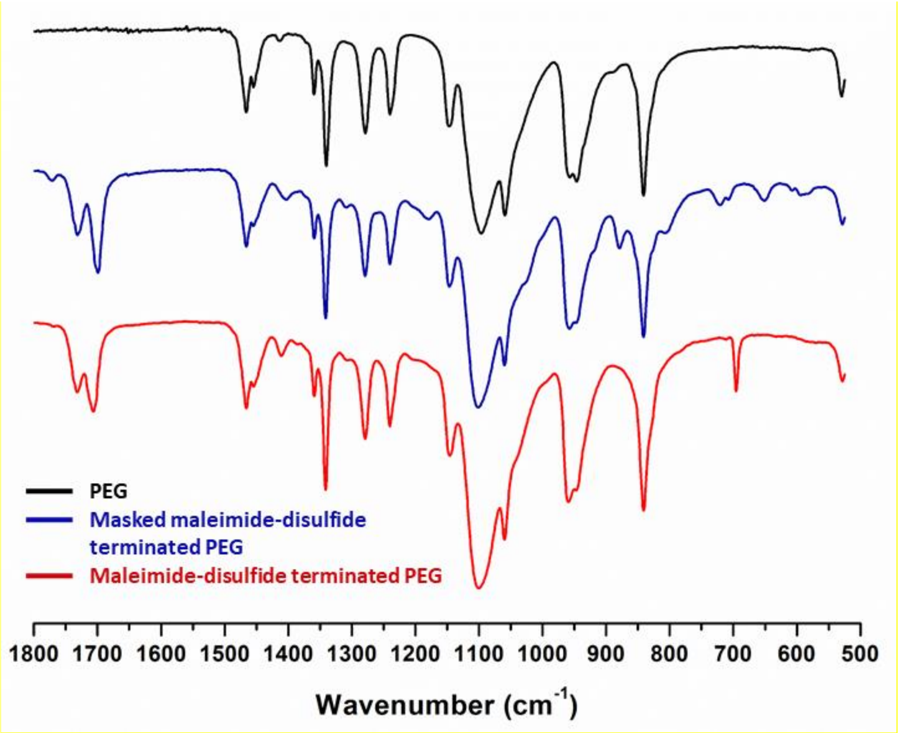

Figure S6. FTIR spectra of PEG, masked maleimide-disulfide terminated PEG, and maleimide-disulfide terminated PEG.

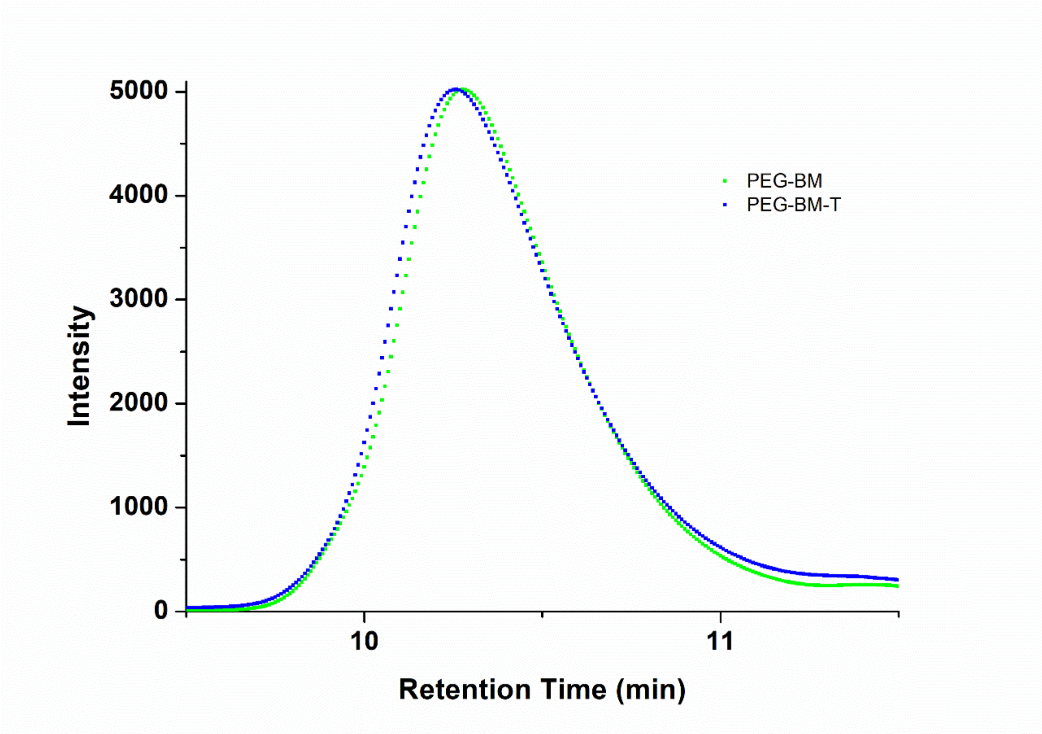

Figure S7. SEC plots of PEG polymers before and after addition of 2-mercaptoethanol.

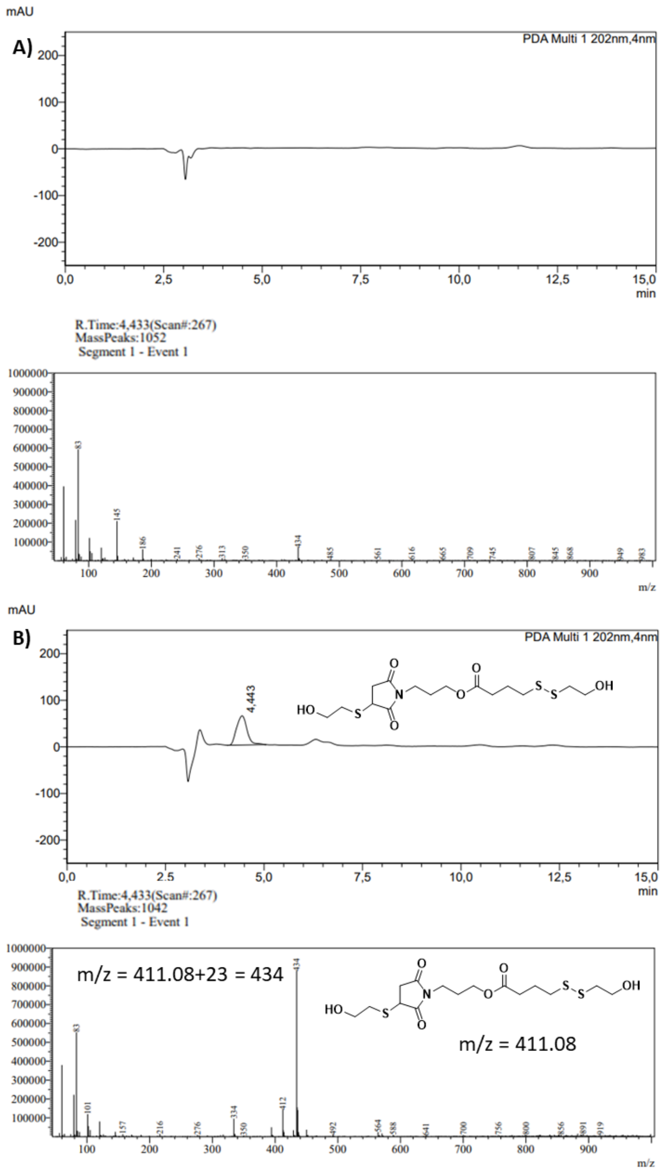

Figure S8. LCMS characterization of the solution of the reaction between maleimide-disulfide terminated PEG with (A) the stoichiometric equivalent of 2-mercaptoethanol and (B) four equivalent of 2-mercaptoethanol.

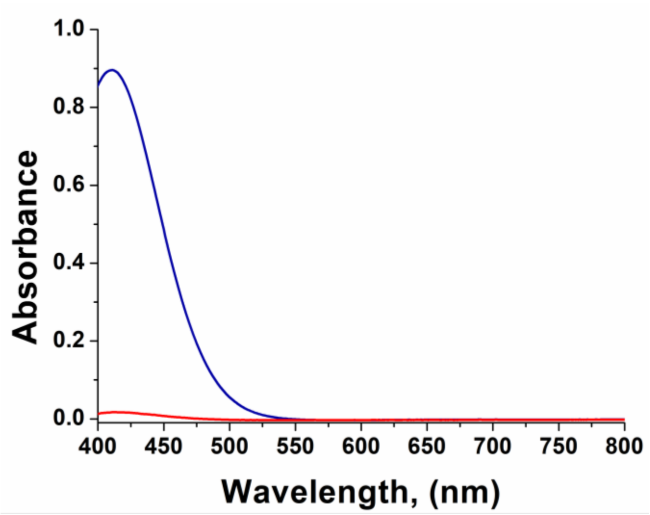

Figure S9. UV-VIS spectra of Ellman's reagent solution incubated with hydrogel (red) and tetra-thiol polymer (blue).

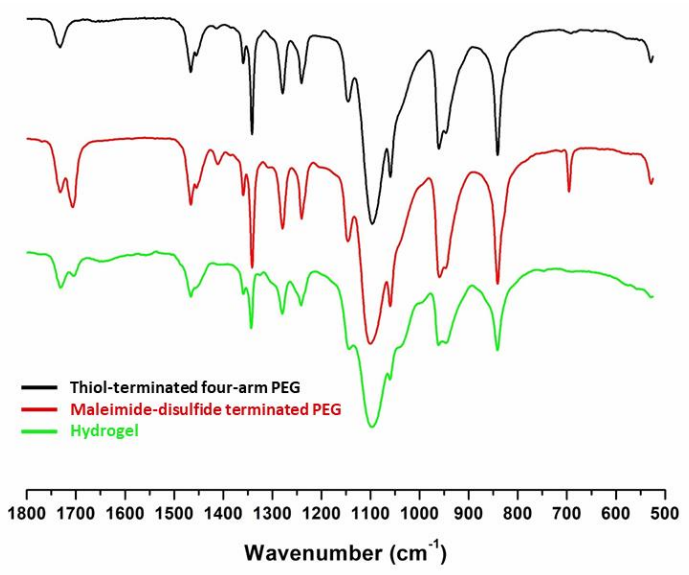

Figure S10. FTIR spectra of thiol-terminated four arm PEG, maleimide-disulfide terminated PEG, and hydrogel.

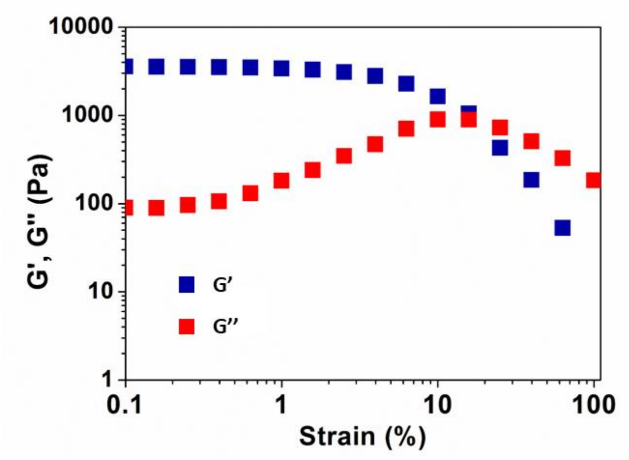

Figure S11. Strain sweep test of the hydrogel.

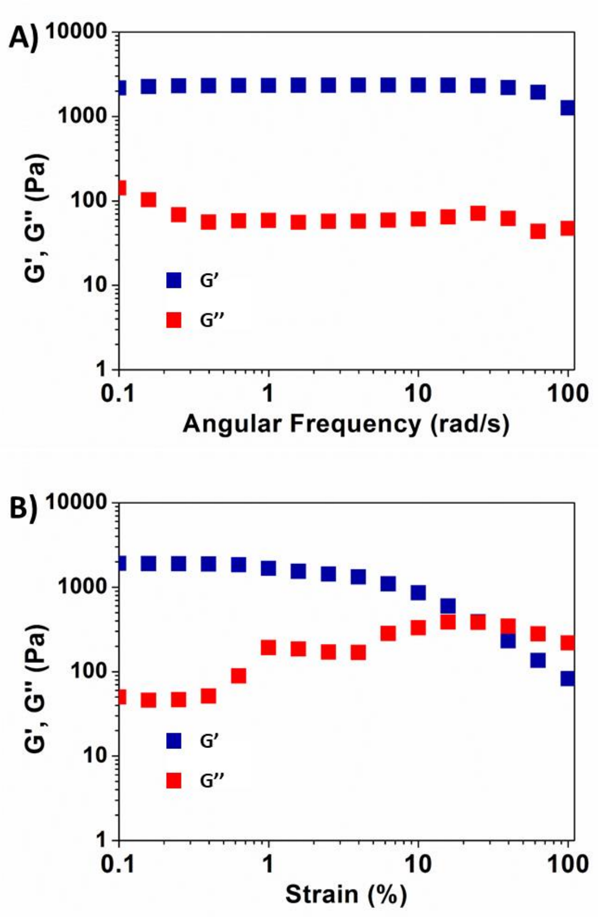

Figure S12. A) Frequency sweep test, and B) strain sweep test of the FITC-BSA loaded hydrogel.

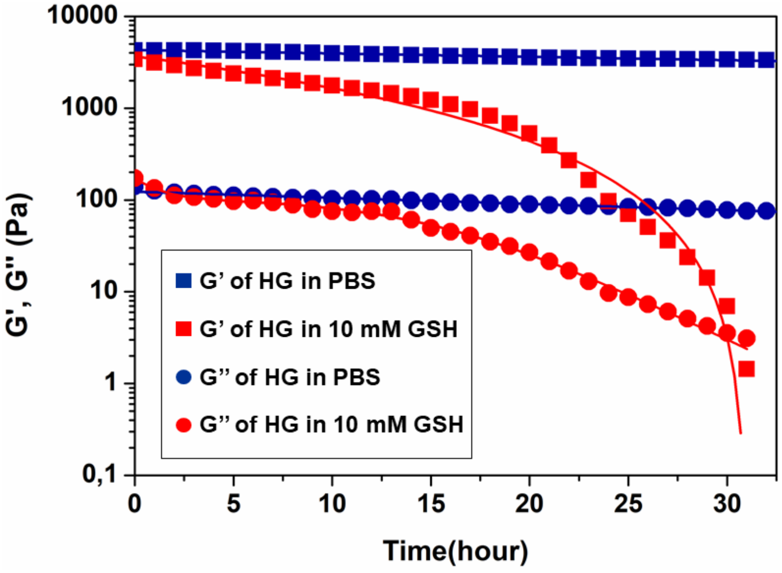

Figure S13. Time sweep test of hydrogel in presence and absence of glutathione (GSH).
